# Effect of congenital adrenal hyperplasia treated by glucocorticoids on plasma metabolome: a machine-learning-based analysis

**DOI:** 10.1038/s41598-020-65897-y

**Published:** 2020-06-01

**Authors:** Lee S. Nguyen, Edi Prifti, Farid Ichou, Monique Leban, Christian Funck-Brentano, Philippe Touraine, Joe-Elie Salem, Anne Bachelot

**Affiliations:** 1 Sorbonne Université, Clinical Investigation Center Paris-Est, CIC 1901, INSERM, AP-HP, Pitié-Salpêtrière University Hospital, Paris, France; 2 CMC Ambroise Paré, RICAP, Neuilly-sur-Seine, France; 3 Institute of Cardiometabolism and Nutrition (ICAN), Integromics, Paris, France; 4 IRD, Sorbonne University, UMMISCO, UMI 209 Paris, France; 5 ICANalytics, Institute of Cardiometabolism and Nutrition (ICAN), Paris, France; 6 Department of Endocrinology and Reproductive Medicine, Sorbonne Université, AP-HP, Pitié-Salpêtrière Hospital, Centre de Référence des Maladies Endocriniennes Rares de la Croissance, Centre de Référence des Pathologies Gynécologiques Rares, ICAN, Paris, France

**Keywords:** Endocrinology, Endocrine system and metabolic diseases, Multihormonal system disorders

## Abstract

Background. Congenital adrenal hyperplasia (CAH) due to 21-hydroxylase deficiency leads to impaired cortisol biosynthesis. Treatment includes glucocorticoid supplementation. We studied the specific metabolomics signatures in CAH patients using two different algorithms. Methods. In a case-control study of CAH patients matched on sex and age with healthy control subjects, two metabolomic analyses were performed: one using MetaboDiff, a validated differential metabolomic analysis tool and the other, using Predomics, a novel machine-learning algorithm. Results. 168 participants were included (84 CAH patients). There was no correlation between plasma cortisol levels during glucocorticoid supplementation and metabolites in CAH patients. Indoleamine 2,3-dioxygenase enzyme activity was correlated with ACTH (rho coefficient = −0.25, p-value = 0.02), in CAH patients but not in controls subjects. Overall, 33 metabolites were significantly altered in CAH patients. Main changes came from: purine and pyrimidine metabolites, branched aminoacids, tricarboxylic acid cycle metabolites and associated pathways (urea, glucose, pentose phosphates). MetaboDiff identified 2 modules that were significantly different between both groups: aminosugar metabolism and purine metabolism. Predomics found several interpretable models which accurately discriminated the two groups (accuracy of 0.86 and AUROC of 0.9). Conclusion. CAH patients and healthy control subjects exhibit significant differences in plasma metabolomes, which may be explained by glucocorticoid supplementation.

## Introduction

Congenital adrenal hyperplasia (CAH) is a group of autosomal recessive diseases due to enzyme deficiencies leading to impaired cortisol biosynthesis and deficiency of the 21-hydroxylase enzyme is its most common form. Main treatment relies on steroid replacement therapy including glucocorticoids (GC) (i.e. hydrocortisone) alone or in association with mineralocorticoids (i.e. fludrocortisone)^1^. Although effective, long-term GC replacement therapy is associated with multiple adverse effects. They include, but are not limited to the appearance of cardiovascular metabolic risk factors (hypertension, insulin-resistance, obesity and dyslipidemia), immunosuppression with infections, psychological disturbances and osteoporosis. Moreover, oral GC intake does not supplement adequately cortisol’s physiological circadian rhythm, and the dosage needed to suppress androgens is usually higher than that needed for substitution only^2^. These elements all contribute to increased health costs and decrease of quality of life and life expectancy^2–6^.

In CAH patients, dosage adjustment of GC routinely consists in assessing clinical symptoms and adrenal androgen levels^7^. Because of a narrow therapeutic index, metabolomic analyses may be suitable to better characterize the effects of this medication and prevent its adverse effects while conserving its benefits. Indeed, pharmacometabolomic studies quantify and analyze circulating metabolites following drug administration, specifically identifying activated pathways^8,9^. Previous metabolomic analyses of CAH patients showed that those requiring a higher dosage of GC presented a different metabolomic profile compared with those who required a lower dosage, with altered free fatty acids, bile acids, and amino acid metabolites pathways^10^. However, no comparison was performed with healthy control subjects, hence, impact of CAH on metabolome remains unclear.

In the present study, we aimed to compare the metabolomic profile of CAH patients treated with GC supplementation, to healthy control subjects, using a machine-learning-based algorithm (*Predomics*)^11^. A previously published package of metabololomic analyses (*MetaboDiff*) was used as quality-controller^12^.

## Results

### Clinical and hormonal differences between CAH and control

Table 1 summarizes clinical and characteristic parameters from this prospective study cohort (n = 168), which included 84 CAH patients and 84 healthy control subjects. Both groups were similar in age, gender proportion, systolic and diastolic blood pressure, history of cardiovascular disease (1.2% vs 7.1%, p = 0.054) and smoking status (active or past represented 27% in both groups). Healthy control subjects were taller than CAH patients (169 vs 163 cm, p < 0.001) and had lower body-mass index (23.1 vs 25.8, p < 0.001). Clinical and biological differences between gender and between groups were analyzed in a previous paper^13^. Among CAH patients, 42 (50%) had classic salt-wasting form, 16 (19.0%) with simple virilizing form and 26 (31.0%) had non-classical form.

**Table 1:** Clinical and hormonal features of control subjects and CAH patients.

	Control (n = 84)			CAH (n = 84)			p-value
	Median	Perc. 25	Perc. 75	Median	Perc. 25	Perc. 75	
Age (years)	27.2	23.4	35.0	28.2	22.6	36.9	0.88
Height (cm)	169.0	163.0	177.0	163.0	157.0	170.0	0.001
Weight (kg)	67.8	60.5	74.0	68.0	57.5	78.0	1.0
BMI	23.07	20.88	25.61	25.8	22.19	29.03	0.001
Systolic blood pressure	112.0	105.0	120.0	110.0	103.0	123.0	0.64
Diastolic blood pressure	70.0	64.0	74.0	68.0	59.0	75.0	0.16
Insulin	5.4	4.0	8.2	8.8	5.8	13.0	<0.001
HOMAIR	1.0	0.8	1.7	1.8	1.1	2.5	0.001
Estradiol (pg/mL)	46.5	27.7	146.0	48.5	30.5	99.0	0.88
Progesterone (ng/mL)	0.8	0.4	1.3	2.0	0.8	6.4	<0.001
FSH (IU/mL)	4.9	3.3	7.6	4.9	2.9	6.5	0.88
LH (IU/mL)	5.7	4.3	9.2	5.2	2.7	7.5	0.22
Androstenedione (ng/mL)	2.3	1.6	3.0	2.8	1.4	5.3	0.17
17-OH-progesterone (ng/mL)	1.6	0.9	2.4	11.9	2.8	29.5	<0.001
Total testosterone (ng/mL)	0.4	0.3	4.5	0.8	0.3	2.9	0.003
SHBG (ng/mL)	56.1	35.3	76.2	46.7	29.9	82.3	0.28
ACTH (pg/mL)	19.5	12.4	29.9	31.3	10.3	67.2	0.009
Renin (pg/mL)	12.8	8.2	17.0	19.5	12.1	41.7	0.001
Aldosterone (pg/mL)	107.0	73.1	148.0	137.0	114.0	264.0	0.09
Cortisol (µg/L)	110	77.5	140	94.85	35.65	144	0.49

Abbreviations: ACTH: Adreno CorticoTrophic Hormone; BMI: body-mass index; FSH: Follicle Stimulating Hormone; HOMAIR: Homeostasic model assessment of insulin resistance; LH: luteinizing hormone; Perc: percentile; SHBG: Sex hormone-binding globulin; 17-OH: 17α-Hydroxyprogesterone.

Compared to healthy control subjects, CAH patients presented significantly higher levels of insulin levels, HOMAIR, 17-OH progesterone, progesterone, total testosterone, ACTH, renin and aldosterone (see Table 1).

### Correlations between hormones and metabolites

Correlations are presented within a heat map provided in Supplementary Figure A. Overall, univariate correlations were not significant below a p-value < 0.001, which makes the following observations uncertain due to the number of tests performed.

Briefly, in CAH patients, 17-OH-progesterone levels correlated with fatty acid metabolites, hexoses (sum of glucose, galactose and fructose) and urea. In healthy control subjects, 17-OH progesterone correlated with arginine and proline.

In both groups, androstenedione levels correlated with succinate, glutamate, fatty acid metabolites, urea cycle, arginine and proline metabolites. Androstenedione also correlated with leucine, isoleucine and valine metabolism pathway in healthy control subjects.

In CAH patients, renin levels correlated with glutamate and lysine metabolites, while it only correlated with tryptophan metabolites in healthy control subjects. There was no association between plasma cortisol levels and metabolic pathways in CAH patients, whereas in control patients, cortisol correlated with lysine, leucine, isoleucine, valine, steroid, nicotinate and nicotinamide metabolites.

Finally, in CAH patients, indoleamine 2,3-dioxygenase (IDO) activity correlated with ACTH (*rho* = −0.25, *p* = 0.02), whereas it was not in control participants. The association between IDO and ACTH in CAH patients was independent from GC supplementation (p < 0.001), after adjustment in multivariable analysis using global linear modeling.

### Metabolic differences between CAH and control patients

**Figure 1:**
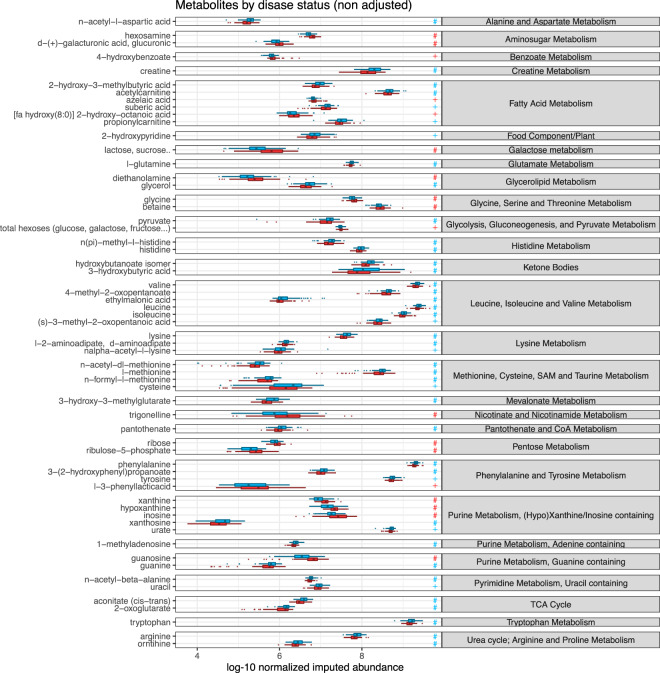
Differences in metabolite abundance between CAH and control patients. Abundance was log-10 transformed before imputation. Legend: blue color represents CAH patients. + sign denotes significant difference in unadjusted analysis only; # denotes significant difference after adjusting for multiple comparisons.

Differences in metabolite abundance between CAH and control patient are presented in Fig. 1 and Supplementary Table A. Overall, between the two groups, 50 metabolites differed significantly (denoted with a ‘+’ symbol in Fig. 1), 33 of which, resisted adjustment for multiple testing (denoted with a ‘#’ symbol in Fig. 1). Main pathways identified included: purine and pyrimidine metabolism, branched aminoacid metabolism, tricarboxylic acid (TCA) cycle and associated pathways (urea, glucose, pentose phosphates).

**Figure 2:**
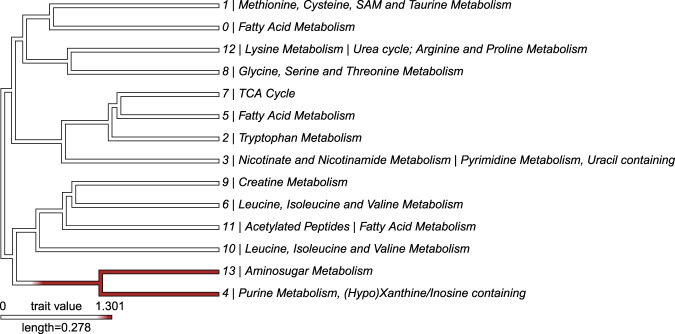
MetaboDiff module visualization diagram with differences between CAH and control patients. Modules are represented as branches of a dendrogram, red color denotes significant difference in module abundance between CAH and control patients.

The comparative metabolomics package “*MetaboDiff”* showed significant differences between the two groups and isolated two modules: module 13 | Aminosugar metabolism and module 4|Purine metabolism, (Hypo)Xanthine/Inosine containing (see Fig. 2).

**Figure 3:**
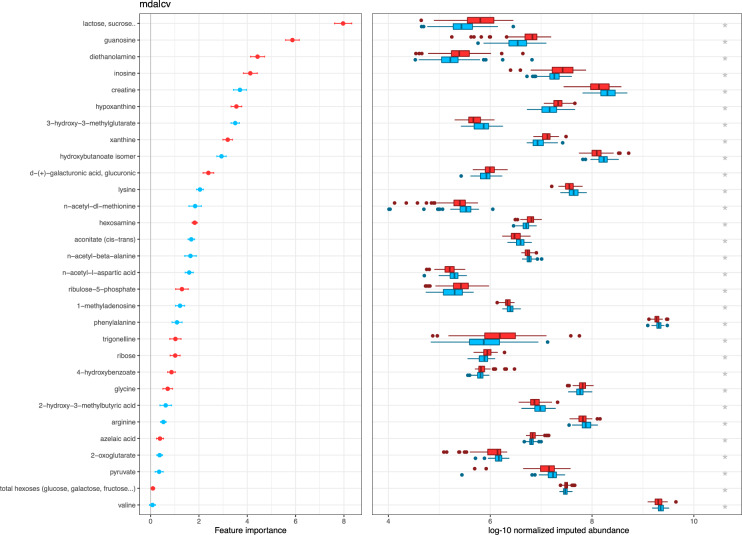
Feature importance of metabolites in models created by Predomics. *Left*: The feature importance (mean decrease accuracy) of the Ter models. Features (rows) are ordered by the average MDA in the three experiments. *Right*: boxplots indicating the distribution of the same metabolites in the two study groups. The blue color indicates enrichment in the CAH group while red in the controls (i.e. in the boxplots, lactose is enriched in the control group). On the left panel, the colours indicate the same concepts also associated with the sign of the features in the TER model (-1 is blue, and 1 is red).

Similarly, the machine-learning based approach identified ternary (Ter) models combining several metabolites that accurately classify CAH and control patients. Metabolites which featured the higher importance in these models included (decreasing order, with featuring importance greater than 2.5): lactose/sucrose, diethanolamine, guanosine, xanthine, creatine, 3-hydroxy-3-methyglutarate, inosine, hydroxybutanoate, hypoxanthine and hexosamine (see Fig. 3).

Moreover, two models illustrating the machine-learning approach were manually generated. A first Ter model combining 4 metabolites (guanosine, inosine, xanthine and creatine) reached an empirical accuracy of 0.82 and area under receiver operator characteristics curve (AUROC) of 0.86 and a second Ter model combining 4 metabolites (guanosine, inosine, xanthine and 3-hydroxy-3-methyglutarate), empirical accuracy was 0.81 and AUROC 0.85.

For comparison purpose, the best automatically generated 4-features Ter model found a combination of xanthine, hydroxybutanoate isomer, n-acetyl-beta-alanine and hexosamine. Its empirical accuracy was 0.86 and AUROC 0.9 (estimated generalized accuracy of the algorithm was 0.74 and AUROC was 0.86).

## Discussion

The main findings of this study are: (i) CAH patients treated with GC express a specific metabolomic signature allowing for metabolomic analyses to accurately discriminate them from healthy control subjects matched on gender and age and (ii) the machine-learning-based approach, *Predomics*, finds concordant results with the differential metabolomics analysis tool *MetaboDiff*, and allows to generate accurate and interpretable models.

Metabolomics analyses showed that CAH patients exhibited a specific metabolic signature. Main altered pathways were those influencing the amount of purine and pyrimidine metabolites, branched amino acids, tricarboxylic acid (TCA) cycle metabolites (urea, glucose, pentose phosphates). The isolated use of GC in healthy control subjects has previously been associated with metabolomics alterations^14^. These alterations were important even for small dosage of GC supplementation and redisposed all the main energetic pathways, including glycolysis, TCA cycle, urea cycle and their connection with fatty acid and amino acids. In the present work, most differences between CAH patients and control subjects lied on these same pathways. A plausible explanation may be that CAH patients may not exhibit other metabolomics alterations than those related to treatment. With similar cortisol levels in both groups, and without any significant correlation between cortisol and metabolic pathway; these findings support the fact that cortisol is not an adequate marker of disease control in this well-supplemented population. Moreover, diurnal cortisol intra-individual variations is well-known factor difficult to predict or correlate.

In this cohort, ACTH was associated with the IDO enzyme activity. IDO catalyzes the reaction degrading tryptophan to kynurenine, passing the rate-limiting step in the pathway which ends to nicotinamide adenine dinucleotide (NAD) biosynthesis^15,16^. ACTH is overexpressed by the pituitary gland in uncontrolled CAH patients, due to a lack of negative feedback^1^. Incidentally, in CAH patients, the most common mutation results in a 21-hydroxylase deficiency, which in humans, may be mimicked by a microsomal reduced NAD phosphate-dependent cytochrome p450 enzyme (POR) deficiency^17^. While POR deficiency also involves several other metabolic pathways, the association found between ACTH and IDO enzyme activity may explain how NAD pathway is altered in both diseases. Remarkably, the association remained statistically significant after adjusting for GC supplementation in multivariable analysis.

All metabolites found by the machine-learning based approach *Predomics*, to discriminate CAH patients from healthy control subjects, were related to pathways known to be influenced by GC treatment. Specifically, purine metabolism (containing xanthine, hypoxanthine, guanosine, inosine) highly discriminated CAH patients from healthy control subjects; followed by aminosugar metabolism (lactose, sucrose, hexosamine), branched aminoacid metabolites (3-hydroxy-3-methylbutyrate) and creatine, mevalonate and ketones metabolism, all which branch into the citrate cycle. Combining purine metabolites to another identified metabolite allowed to manually generate simple models with only four features, which showed high accuracy and discrimination, as compared to automatically selected models.

### Limitations

We acknowledge several limitations in this study. Although the number of patients was relatively high for this rare disease, the sample size still was not very large and results might differ in a larger population of CAH patients. Second, correlations were moderate which may be due to confounding factors altering levels of circulating metabolites. The external validity of these results relies in part, on the fact that they comfort biochemical mechanisms proven experimentally decades ago for some of them (i.e. glucocorticoid-induced purine metabolism)^18^, or in the last five years, using advanced metabolomic tools, for others (i.e. metabolomic profiling of healthy subjects supplemented in GC)^14^. Regarding statistical analyses, we performed state-of-art data-processing techniques, p-value correction methods using Bonferroni-Hochberg procedure and stringent data filtering; we also omitted variables that were associated with medication intake to avoid false-positives and applied non-parametric statistics which are robust to normality distributions but at the expense of power. The machine-learning-based approach used here minimizes the sum of a cost function and regularizes L1 norm for sparsity, under a constraint on the unary value of the linear model that predicts classes. Besides being relatively simple and interpretable, the generated models are robust to generalization and offer importance scoring of the features.

## Conclusion

CAH patients and healthy control subjects display significantly different plasma metabolomes signatures. Main differences may be explained by GC supplementation of CAH patients only. Predomics identified such simple signatures allowing for accurate discrimination between CAH and healthy control subjects.

## Methods

### Study design

This study is ancillary to the *Cardiovascular Risk Profile in Patients With Congenital Adrenal Hyperplasia* (CARDIOHCS) study (clinicaltrials.gov identifier **NCT01807364**), a multicenter prospective observational case-control study comparing early cardiovascular damage in adult men and women with CAH due to 21α-hydroxylase deficiency and healthy control subjects^13,19,20^. All participants provided written informed consent to participate, and the study was approved by each hospital ethics committee including Pitie-Salpetriere University Hospital.

### Study population

Patients with CAH and healthy subjects were all assessed at the Clinical Investigation Center Paris-Est (CIC-1901, Pitié-Salpêtrière University Hospital, Paris, France). Eighty-four young adults (58 women and 26 men) with CAH and 84 control subjects matched for sex, age, and smoking status were prospectively included in the CARDIOHCS study between May 2011 and August 2015^13,19,20^. The adults with CAH included in the study, were diagnosed during childhood and confirmed by genetic testing of 21α-hydroxylase deficiency. Exclusion criteria for CAH and healthy subjects were: previous history of cardiovascular disease, use of combined contraceptives within 1 month of data collection, and current pregnancy. Patients with CAH were treated, as needed by their standard of care, with hydrocortisone or dexamethasone, and fludrocortisone. All patients required to be fasting for at least 12 hours prior to blood sampling, and to refrain from coffee-containing products for at least 24 hours.

### Study objectives

The main objective of this ancillary study was to characterize the metabolomic profile of CAH patients compared to that of matched healthy controls using untargeted approaches. To this aim, two analytic methods were performed.

### Hormone analyses

Blood samples for the determination of serum concentrations of 17-OH progesterone, progesterone, estradiol, total testosterone, aldosterone, renin, androstenedione, sex hormone–binding globulin (SHBG), adrenocorticotropic hormone (ACTH), follicle-stimulating hormone (FSH), luteinizing hormone (LH) were collected in dry tubes and further assayed in the immunology laboratory of Pitié-Salpêtrière University Hospital. Estradiol, progesterone, SHBG and testosterone were assayed using chemiluminescence (Modular-E170; Roche, Mannheim, Germany) and 17-OH progesterone by radioimmunoassay (KIP1409; DIAsource ImmunoAssays, Louvain-la-Neuve, Belgium). Cortisol was sampled concomitantly with the other hormones, more than 8 hours after last intake of corticosteroids in CAH patients and after 12 hours of fasting in healthy subjects. Baseline hormonal measurements were assessed in all subjects, under their regular treatment for CAH patients.

### Metabolomic analyses preparations

Eight volumes of frozen acetonitrile (−20 °C) containing internal standard (labelled mix of amino acids at 12.5 μg/mL) were added to 50 μL serum samples and vortexed. Samples were sonicated for 15 minutes and centrifuged for 2 minutes at 10.000 × g and at 4 °C. Then, centrifuged samples were incubated at 4 °C during one hour for slow protein precipitation. Samples were then centrifuged at 20.000 × g at 4 °C and supernatants were transferred to another series of tubes and then dried out and frozen at −80 °C until the liquid chromatography-mass spectrometry (LC-MS) analyses. Samples were reconstituted based on starting mobile phase composition of the chromatographic method (water/acetonitrile (99:1; v-v) containing 0.1% of formic acid). Reconstituted samples were, then, centrifuged and transferred to vials before LC-MS analyses.

### Ultra-performance Liquid Chromatography-Mass Spectrometry (UPLC-MS) analyses of serum samples

Metabolite profiling analysis was carried out on a UPLC Waters Acquity (Waters Corp, Saint-Quentin-en-Yvelines, France) coupled to a hydrid Orbitrap based instrument, a Q-Exactive (Thermo Fisher Scientific, Illkirch, France). LC-MS analyses was made in full scan positive and negative mode with a resolution of 70 000 (FWHM) and a scan range of m/z 50–750. Mass spectrometer was systematically calibrated before LC-MS analyses in both ion polarity modes with the Pierce calibration solution.

LC-MS analyses were performed using a modification of the method of Garali *et al*. to screen microbiota-derived metabolites^21^. Briefly, a ‘PFPP column’ Discovery HS F5-PFPP column, 3 μm, 2.1 × 150 mm (Sigma, SUPELCO, Saint Quentin Fallavier, France) kept at 35 °C. Injection volume and autosampler temperatures were set to 10 μl and 5 °C in both column systems, respectively. Flow rate was set to 0.250 mL/min and the mobile phase consisted of 0.1% of formic acid in water (A) and 0.1% of formic acid in acetonitrile (B) during 28 minutes.

### Untargeted metabolomics data analyses

Processing steps of MS data, including peak picking, peak grouping, retention time correction, and annotation of isotopes and adducts, were performed using XCMS R package with CentWave algorithm and CAMERA tools implemented in R software and the galaxy workflow4metabolomics^22–24^. Processing of LC-MS data were analyzed based on standard protocols^25,26^ and resulted in a datamatrix in which each metabolomic feature was characterized by a retention time (RT), mass to charge ratio (m/z), and its corresponding intensities for each sample and the isotope-adduct annotation from CAMERA tool.

Metabolomics data matrix was filtered, normalized, curated and log-10 transformed based on quality assurance (QA) strategy^27,28^. Peaks with more than 30% of missing values were discarded. Metabolites from the Xenobiotics category were also discarded. Principal component analysis (PCA) was also plotted to assess the absence of technical drift during data acquisition process (data not shown). Robust features were annotated based on their mass to charge ratio (m/z) and retention time (RT) using public, and ‘in-house’ databases then confirmed based on MS/MS experiments. Following these processing and annotation steps, 140 features met the acceptance criteria. Moreover, indoleamine 2,3 dioxygenase (IDO) enzyme activity was computed as the abundance ratio of kynurenine over tryptophan.

### Statistical analysis

Continuous variables are presented as median (interquartile range) and categorical variables as number (proportions). For enrichment and statistical analyses of the metabolomics data the *MetaboDiff* R package (v 0.9.3) was used^29^. Briefly, the *MetaboDiff* analysis consists on different steps, including annotation, imputing missing data by k-nearest neighbor approach and removal of metabolites with at least 40% of missing data. Data are then renormalized using variance stabilizing normalization to ensure that variance remains nearly constant over the measured spectrum^30^. To derive meaningful subpathways that are enriched between groups, *MetaboDiff* generates a metabolic correlation network, which offers the possibility to integrate external information such as pathway information^12^. Module significance can be determined as the average absolute metabolite significance measure. The *Predomics* approach, based on specific machine-learning models and genetic algorithm, searches a few sparse models in a very large combinatorial space. The best ternary models is then selected by applying a penalty on the number of features used – for each feature added the accuracy is decreased by a value of 1/100. A feature importance score is computed during the cross validation^11^. The resulting data were used to assess differential abundance between patients and controls and using unpaired non-parametric tests (Mann-Whitney). Associations with clinical numerical variables were tested using Spearman correlations. P-values were adjusted for multiple testing using the Benjamini-Hochberg method^31^. Prediction analyses were performed using the *Predomics* package searching for ternary models with the beam-search algorithm using default parameters^11^. Here we used the *MetaboDiff* imputed data prior to normalization for interpretability reasons. Cross-validation was performed using a 20-times 5-fold configuration and the sparsity penalization coefficient was set to 1%.

### Statement

All described methods were carried out with relevant guidelines and regulations.

## Supplementary information

Supplementary information.
